# A Female Newborn With Occipital Encephalocele and a Hypoplastic Right Ventricle Secondary to Tricuspid and Pulmonary Atresia: A Case Report

**DOI:** 10.7759/cureus.55917

**Published:** 2024-03-10

**Authors:** Alexandria Sobczak, Alyson Skelly, Hemangi Patel, Randy Felber, Christine DiLeo

**Affiliations:** 1 Medicine, Dr. Kiran C. Patel College of Osteopathic Medicine, Nova Southeastern University, Fort Lauderdale, USA; 2 Obstetrics and Gynecology, Wellington Regional Medical Center, Wellington, USA

**Keywords:** neural tube defects (ntds), congenital cardiac malformation, tricuspid atresia, hypoplastic right ventricle, occipital encephalocele

## Abstract

Encephaloceles and severe cardiac malformations are rare presentations in a newborn. The mechanism of these congenital abnormalities is relatively unknown, but it is hypothesized to be related to genetic, environmental, and maternal risk factors. This case report describes a newborn with an occipital encephalocele associated with severe right ventricular hypoplasia secondary to tricuspid and pulmonary atresia. The patient’s maternal risk factors included obesity, type 2 diabetes mellitus, and everyday tobacco use during pregnancy. Education on preconception planning, management, and counseling is essential as a preventative measure in fetal development and is further emphasized in this case.

## Introduction

There are many congenital malformations of the CNS that arise from the disruption in the formation of the neural tube. The neural tube is formed by the fusion of ectodermal folds during the embryological development of the fetus. It leads to the creation of the brain and spinal cord, mainly during the 3rd and 4th week of pregnancy [1]. When there is a lack of closure and failure of the ectoderm to separate and form the neuroectoderm, there is a defect in the skull that forms with herniation of the brain tissue [1]. Encephaloceles can affect any part of the skull, but 75% of cases affect the occipital area [1].

The etiology of encephalocele is unknown but can be due to many genetic and environmental factors. The genetic causes include chromosomal abnormalities, and the environmental causes include deficits in maternal nutrition, toxins, and various infections [1]. During the process of neural tube development in the fetus, adequate levels of vitamin B12, folate, and homocysteine is essential to reduce the risk of newborn malformations. Also, it is imperative to avoid illnesses, specifically toxoplasma, rubella, cytomegalovirus, herpes simplex virus (TORCH) infections because they are involved in many cases of encephaloceles [1]. Lastly, it is important to avoid any teratogens such as tobacco and alcohol. 

Congenital heart diseases occur while the fetus is developing in utero, with one in 100 children having genetic or chromosomal abnormalities related to the defect [2]. Further consumption of teratogens such as alcohol and tobacco, TORCH infections, especially in the first trimester of pregnancy, and lack of nutrition in pregnancy are known risk factors for congenital heart disease in children [2]. Similarly, fetal ventriculomegaly, defined as ventricular enlargement, is diagnosed in utero and is known as one of the most common fetal anomalies with etiologies similar to encephalocele and cardiac abnormality causes, including genetic, environmental, and TORCH infection exposure [3]. 

There are many maternal risk factors leading to congenital malformations such as type 2 diabetes mellitus and tobacco use during pregnancy as seen in this case presentation. We present the case of a 30-year-old female presenting for a cesarean section at 37 weeks gestation due to significant fetal anomalies comprised of cardiac abnormalities and encephalocele. 

## Case presentation

A 30-year-old female gravida 5, para 1, with one term, zero preterm, three abortions, and one living child (G5P1031) presented for a scheduled cesarean section at 37 weeks gestation. Her pregnancy was complicated by maternal type 2 diabetes mellitus, maternal obesity with a body mass index (BMI) of 42.5 kg/m^2^, daily tobacco use, and cholestasis of pregnancy. Her history is significant for fetal macrosomia in a prior pregnancy delivered via cesarean section. Due to patient refusal, the tetanus-diphtheria-pertussis and the seasonal influenza immunizations were not given during the course of the pregnancy. Maternal prenatal visits were completed as scheduled with her first prenatal visit occurring at 12 weeks. 

At 20 weeks gestational age, a level II ultrasound was performed. On examination, the cranium was seen to be abnormally shaped with depressed frontal bones. A posterior cranial bone defect and herniated fluid-filled cysts were present. A complex cardiac defect was also noted on ultrasound. The left ventricle was noted to be larger than the right, and the tricuspid valve looked atretic. 

A follow-up to the level II ultrasound was performed at 22 and 26 weeks. On both ultrasound examinations, cardiac activity was present with fetal heart rates (FHR) of 155 beats per minute (bpm) and 137 bpm which were within normal limits. On a four-chamber view, a hypoplastic right ventricle was present and outflow through the right side of the heart was not seen, consistent with pulmonary and tricuspid atresia. The persistence of the abnormal shape of the cranium and abnormal posterior fossa were consistent with an encephalocele. 

Extensive genetic testing was done throughout the pregnancy, beginning at 15 weeks gestation. Amniocentesis performed at 20 weeks showed low risk for chromosomal abnormalities including, but not limited to, trisomy 13, trisomy 18, trisomy 21, monosomy X, and triploidy.

During the final transabdominal scan at 33 weeks gestation, the fetus’ occipital lesion measured 5.36 cm x 3.01 cm x 4.27 cm. Ventriculomegaly was present with lateral ventricles dilated to 12.8 mm on the right and 10.4 mm on the left with the 4th ventricle also dilated to 2.1 cm x 1.1 cm. 

An echocardiogram was performed two days prior to the scheduled cesarean, and a complex single ventricle anatomy with pulmonary atresia was still noted. Although a magnetic resonance imaging (MRI) was recommended to better visualize the cranial abnormalities, the patient declined.

On maternal arrival for scheduled cesarean, her vitals were heart rate (HR), 88 bpm; systolic blood pressure (SBP), 116 mmHg; diastolic blood pressure (DBP), 67 mmHg; SpO2, 97% saturation; height, 160 cm; weight, 240 lbs; and BMI, 42.51 kg/m^2^. Her medications included diphenhydramine 50 mg capsule twice a day, ursodiol 300 mg capsule once a day, metformin 500 mg capsule once a day, and a daily prenatal vitamin. Prenatal vitamins were discussed at the first prenatal visit, but it is unknown exactly when she began taking them. 

Low transverse cesarean section was performed as planned and a viable female infant weighing 7 lbs, 12 oz was delivered. Infant appearance, pulse, grimace, activity, respiration (APGAR) scores were 7 at one minute and 8 at five minutes. At delivery, the infant was active, was spontaneously breathing, and had a heart rate greater than 100 bpm. The infant was transferred to the neonatal intensive care unit (NICU) in stable medical condition on room air. 

**Figure 1:**
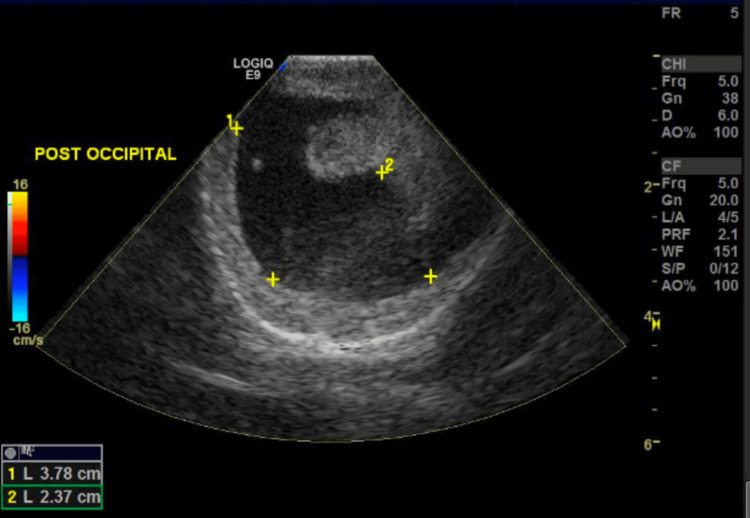
Transcranial grayscale ultrasound image of an occipital lesion consistent with an encephalocele

**Figure 2:**
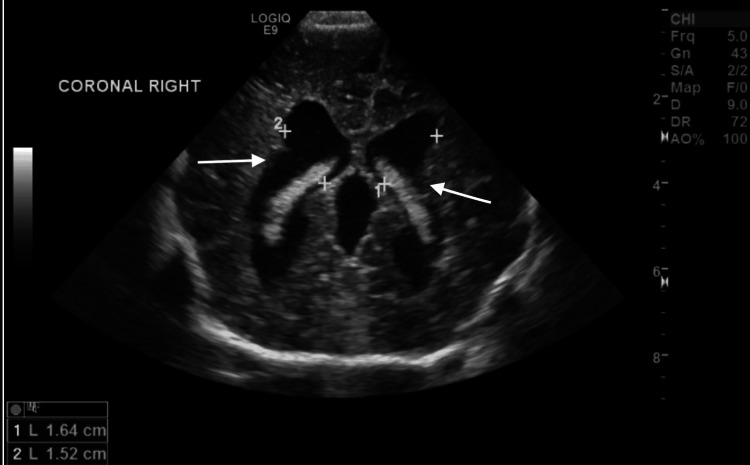
Ventriculomegaly of the lateral ventricles identified on ultrasound Ventriculomegaly of the lateral ventricles as indicated by the arrows

On further examination, a 5 x 5 x 3 cm cystic swelling was noted in the posterior occipital region with skin covering the area. A postnatal head ultrasound was consistent with an encephalocele (Figure 1). Postnatal ultrasound also depicted ventriculomegaly of the lateral ventricles with the right lateral ventricle measuring approximately 1.6 cm and the left 1.5 cm in diameter (Figure 2). Additionally, a grade 2-3 systolic murmur was present, and postnatal echocardiogram displayed severe right ventricular hypoplasia with tricuspid and pulmonary atresia (Video 1). 

**Video 1:** Pediatric transthoracic echocardiogram of the patient's congenital cardiac abnormality depicting pulmonary atresia

The patient was started on prostaglandins to maintain ductus arteriosus patency. Prostaglandin administration was foreseen prior to delivery due to the complex cardiac abnormalities seen on imaging. Shortly after, the patient required intubation for central apnea. Patient was monitored closely and caffeine was close on hand incase of continuous apnea. Umbilical artery and venous catheters were placed for lab monitoring and medication management. Postnatal MRI was not performed due to the change in patient status to critical and subsequent transfer that evening to a higher-level children’s hospital for further specialized management.

## Discussion

An encephalocele is an exceedingly rare neural tube defect in which the brain matter and meninges protrude outside of the skull. It occurs in 1 in 10,000 newborns [4]. Encephaloceles located in the occipital region carry a worse prognosis compared to those protruding from the frontal area [4]. The amount of brain tissue within the encephalocele also plays a major role in prognosis. Of those who survive, 52% have some form of disability [4]. Congenital heart defects (CHDs), on the other hand, affect roughly 40,000 newborns, or 1% of births annually [5]. Congenital cardiac abnormalities are the leading cause of infant death. According to a study cited by the Centers for Disease Control and Prevention, CHDs cause approximately 4.2% of neonatal demise [5]. Patients with a complex CHD approximately have a 75% survival rate in the first year of life and a 69% survival rate at 18 years old [5]. Of these individuals, over 50% have some form of developmental impairment [5]. While these abnormalities are rare, both encephaloceles and CHDs are associated with high morbidity and mortality rates in newborns. 

The maternal history, including obesity, type 2 diabetes mellitus, and everyday tobacco use during pregnancy, is a risk factor for these congenital birth defects. Folic acid is essential for the normal fetal cardiac and central nervous system development. A deficiency in folic acid has been associated with both neural tube defects and CHDs [6]. Tobacco use has been shown to be negatively associated with most folate biomarkers, indicating that cigarette smoking reduces serum levels of folic acid, increasing the risk for neural tube defects [7]. It is well-known that diabetic women are at increased risk for both neural tube defects and CHDs. [8]. Increased maternal BMI, as noted in our patient with a BMI of 42.41kg/m^2^, has also been correlated to fetal cardiac abnormalities [9].

The current US Preventive Services Task Force (USPSTF) recommendation is that all persons planning to become pregnant should take a daily supplement containing 0.4 to 0.8 mg of folic acid [10]. It has been shown that higher doses of folic acid (4mg) prevent reduction in fetal body size [11] and optimize brain growth [12] among infants of women who smoke tobacco during pregnancy [11]. Further research is needed to explore the effects of high-dose folic acid in specific populations, such as smokers and individuals with diabetes to reduce the risk of CHDs. 

The risks of obesity, diabetes, and tobacco use during pregnancy should be presented from both the maternal and fetal perspectives and involve a multidisciplinary team including obstetricians, endocrinologists, behavioral specialists, and dieticians [13].

## Conclusions

Encephaloceles are congenital anomalies associated with neural tube defects during early fetal development. The exact cause is relatively unknown, but the incidence is associated with genetic, environmental, and maternal risk factors. Congenital heart diseases are abnormalities of the heart’s structure and function that are present at birth. We discussed a case involving a 30-year-old female with maternal complications leading to the birth of a newborn with a complicated cardiac abnormality and encephalocele. The cause of encephalocele is relatively unknown; however, there may be a correlation between an encephalocele and cardiac abnormalities as seen in this newborn with severe right ventricular hypoplasia with tricuspid and pulmonary atresia. The purpose of this case was to highlight how maternal risk factors such as obesity, maternal diabetes, and tobacco use during pregnancy may impact the fetus. Also, it was to emphasize the importance of preconception planning, management, and counseling as a preventative measure in the development of fetal abnormalities and complications in the obstetric population.
